# Huangqin-Tang Ameliorates TNBS-Induced Colitis by Regulating Effector and Regulatory CD4^+^ T Cells

**DOI:** 10.1155/2015/102021

**Published:** 2015-08-04

**Authors:** Ying Zou, Wen-Yang Li, Zheng Wan, Bing Zhao, Zhi-Wei He, Zhu-Guo Wu, Guo-Liang Huang, Jian Wang, Bin-Bin Li, Yang-Jia Lu, Cong-Cong Ding, Hong-Gang Chi, Xue-Bao Zheng

**Affiliations:** ^1^Department of Traditional Chinese Medicine, Second Clinical Medical College, Guangdong Medical College, Dongguan 523808, China; ^2^China-America Cancer Research Institute, Guangdong Medical College, Dongguan 523808, China; ^3^Second Clinical Medical College, Guangdong Medical College, Dongguan 523808, China

## Abstract

Huangqin-Tang decoction (HQT) is a classic traditional Chinese herbal formulation that is widely used to ameliorate the symptoms of gastrointestinal disorders, including inflammatory bowel disease (IBD). This study was designed to investigate the therapeutic potential and immunological regulatory activity of HQT in experimental colitis in rats. Using an animal model of colitis by intrarectally administering 2,4,6-trinitrobenzenesulfonic acid (TNBS), we found that administration of HQT significantly inhibited the severity of TNBS-induced colitis in a dose-dependent manner. In addition, treatment with HQT produced better results than that with mesalazine, as shown by improvedweight loss bleeding and diarrhoea scores, colon length, and intestinal inflammation. As for potential immunological regulation of HQT action, the percentages of Th1 and Th17 cells were reduced, but those Th2 and Treg cells were enhanced in LPMCs after HQT treatment. Additionally, HQT lowered the levels of Th1/Th17-associated cytokines but increased production of Th2/Treg-associated cytokines in the colon and MLNs. Furthermore, we observed a remarkable suppression of the Th1/Th17-associated transcription factors T-bet and ROR-*γ*t. However, expression levels of the Th2/Treg-associated transcription factors GATA-3 and Foxp3 were enhanced during treatment with HQT. Our results suggest that HQT has the therapeutic potential to ameliorate TNBS-induced colitis symptoms. This protective effect is possibly mediated by its effects on CD4^+^ T cells subsets.

## 1. Introduction

Human inflammatory bowel disease (IBD) comprises the two related chronic, relapsing inflammatory disorders, Crohn's disease (CD) and ulcerative colitis (UC) [1]. Although the detailed etiology and pathogenesis of IBD remain uncertain, recent experimental and clinical studies have suggested that the dysregulation of mucosal CD4^+^ T cells, which contributes to intestinal inflammation and mucosal barrier destruction, is one of the most important aspects of the pathogenesis [2, 3].

Among a variety of inflammatory cells in the gut, both effector CD4^+^ T helper (Th) cells and regulatory CD4^+^ T (Treg) cells are important in IBD, as they regulate pro/anti-inflammatory cytokine production [4]. In general, naive CD4^+^ T cells can be divided into one of several lineages of Th cells, including Th1, Th2, and Th17, which vary in their cytokine production and function [5]. Classically, CD is thought to be caused by a deregulated Th1 inflammatory response, while UC has historically been considered a Th2-mediated disease. Th17 is a new subtype of effector Th cells that has been reported to play a key pathogenic role in chronic inflammatory conditions, including IBD [6]. Treg cells are a specialized population of CD4^+^ T cells that act as dedicated mediators to dampen inflammatory responses and prevent autoimmunity. Several studies have demonstrated an inadequate Treg cells responses in the face of an overly exuberant Th1, Th2, and Th17 cells response, resulting in the breakdown of intestinal homeostasis and profound acceleration of the perpetuation of IBD [7]. Given the key role of CD4^+^ T cells subsets in intestinal inflammation, therapeutics targeting these aberrant CD4^+^ T cells responses is already under development and are promising treatments for IBD and other inflammatory diseases.

The mainstays of current IBD treatments involve the use of corticosteroids, immunomodulators, and biologic agents targeting specific cytokines. Although these drugs are conventional therapeutics, most of these treatments are still being used with reluctance due to the high cost, toxic side effects, and uncertainty about long-term safety [8–10]. Consequently, many patients turn to alternative strategies, including traditional plant-based remedies.

Huangqin-Tang decoction (HQT) is a classic traditional Chinese herbal formulation consisting of 4 components: the roots of* Scutellaria baicalensis* Georgi (scute),* Glycyrrhiza uralensis* Fisch. (licorice),* Paeonia lactiflora* Pall. (peony), and the fruit of* Ziziphus jujuba* Mill. (Chinese date). HQT has been used for nearly 1800 years in traditional Chinese medicine to treat common gastrointestinal distress, such as diarrhoea, abdominal spasms, fever, headache, vomiting, nausea, extreme thirst, and subcardiac distention [11]. Although HQT is also significantly protective in the treatment of IBD in Chinese clinical application, further clinical evidence and definitive mechanisms of action that demonstrate the role of HQT in gastrointestinal diseases are still lacking. Therefore, the aim of this study was to investigate the contribution of HQT to the amelioration of colitis and CD4^+^ T cells immune homeostasis in 2,4,6-trinitrobenzenesulfonic acid- (TNBS-) induced acute colitis.

## 2. Materials and Methods

### 2.1. Rats

Sprague-Dawley rats, weighing 200–250 g, were purchased from the Experimental Animal Center of Guangdong Province (Guangzhou, China). Rats were provided a standard rat chow and water in a controlled room (temperature, 22–24°C; humidity, 70–75%; and a 12 h/12 h light and dark cycle). The animal studies were conducted under protocols approved by the Ethics Committee for Animal Experiments of Southern Medical University. The rats were paired with age-matched controls.

### 2.2. Induction of TNBS-Induced Colitis and Treatment

Colitis was induced with a single intracolonic application of TNBS, as described previously [12]. Briefly, overnight-fasted mice were treated under anesthesia with 30 mg/kg TNBS (Sigma-Aldrich) dissolved in 0.25 mL of 50% alcohol via intrarectal injection using a polyethylene catheter (2 mm in outer diameter), with 0.9% saline treatment as a control. The ingredients of HQT included 9 g of* Scutellaria baicalensis* Georgi (scute), 6 g of* Paeonia lactiflora* Pall. (peony), 6 g of* Glycyrrhiza uralensis* Fisch. (licorice), and 6 g of* Ziziphus jujuba* Mill. (Chinese date). All herb formula granules (1 g extract = 10 g crude herb) were provided by E-Fong Pharmaceutical co., Ltd. (Guangzhou, GD, China) and administered at doses of 30 mg/kg, 60 mg/kg, and 120 mg/kg of body weight, dissolved in distilled water. Mesalazine (500 mg/pack) was used at a dose of 100 mg/kg as a vehicle control and purchased from Ethypharm (Houdan, France). HQT and mesalazine were administered by oral gavage twice daily for one week starting from 24 h after colitis induction. Control rats had free access to tap water.

### 2.3. Clinical Assessment of Colitis

Body weight, diarrhoea scores, and bleeding scores were assessed daily as described [13]. Weight changes were calculated as percent difference relative to original body weight. Stool consistency was scored as follows: 0, well-formed pellets; 2, pasty and semiformed stools that do not adhere to the anus; and 4, diarrhoea that remained adhesive to the anus. Fecal blood was scored as follows: 0, no blood by hemoccult test; 2, positive hemoccult; and 4, gross bleeding.

### 2.4. Macroscopic Evaluation

The colon was removed and opened longitudinally, and the colon length and macroscopic damage were assessed immediately by an independent observer blinded to the identity of treatments. The macroscopic score was assigned by examining an 8 cm distal portion of the rat colon and utilizing a 0–4 scale with some modifications from that used previously [14]: 0, no macroscopic changes; 1, hyperemia and edema without ulcers; 2, hyperemia and edema with small linear ulcers or petechiae; 3, hyperemia and edema with wide ulcers and necrosis and/or adhesions; 4, hyperemia and edema with megacolon, stenosis, and/or perforation.

### 2.5. Histology Scoring

For histological examination, colonic tissue was fixed in 10% formalin, dehydrated, paraffin embedded, processed, sliced into 4 *μ*m thick sections, and stained with hematoxylin and eosin (H&E). All the slides were read and scored by a blinded pathologist. The microscopic damage in the colon was assessed on a 0–3 scale as described by Dieleman et al. [15] as follows: (1) severity of inflammation (0, none; 1, mild; 2, moderate; and 3, severe); (2) extent of inflammation (0, none; 1, mucosal; 2, mucosal and submucosal; and 3, transmural); (3) crypt damage (0, none; 1, basal third damaged; 2, basal two-thirds damaged; 3, crypt loss with present surface epithelium; and 4, crypt and surface epithelium loss). The average of the three histology scores was used for statistical analysis.

### 2.6. Myeloperoxidase (MPO) Activity Assay

MPO activity was measured according to the method described previously [16]. Each segment was weighed, chopped, and then homogenized in a potassium phosphate buffer (50 mM, pH 6.0) containing 5% hexadecyl trimethyl ammonium bromide (HTAB) and 0.336% EDTA (9 mL/mg tissue) for 30 s. The colon homogenates were subjected to 3 cycles of freezing/thawing, 30 s of sonication, and centrifugation at 13,000 ×g for 15 min at 4°C. Then, 0.167 mg/mL o-dianisidine dihydrochloride (Sigma-Aldrich) and 0.0005% H_2_O_2_ in 200 *μ*L of phosphate buffer (pH 6.0) were added to the supernatant, and the absorbance rate was monitored at 490 nm.

### 2.7. Preparation of Lamina Propria Mononuclear Cells (LPMCs)

LPMCs were isolated using a modified method as previously described [17]. Briefly, the intestinal mucosa was washed in complete Hank's balanced salt solution (HBSS) without Ca^2+^ and Mg^2+^, cut into 5 mm pieces, and incubated in medium containing 5 mM EDTA (Sigma-Aldrich, St. Louis, Missouri, USA) and 1 mM DTT (Sigma-Aldrich) at 37°C for 30 min until all crypts and individual epithelial cells were removed. The tissues were digested further in RPMI 1640 (GIBCO Laboratories, Grand Island, NY, USA) containing 10% fetal calf serum (FCS, HyClone, Logan, UT, USA), 0.15 g of collagenase type IV (Sigma-Aldrich), and 0.025 g of DNase I (Sigma-Aldrich) in a shaking incubator at 37°C. The tissue slurry was then passed through a 70 *μ*m cell strainer to remove undigested tissue pieces, centrifuged, and resuspended in a 40–60% Percoll solution (Amersham Biosciences, Piscataway, NJ, USA) density gradient. Cells were frozen in liquid nitrogen for storage until analysis at a concentration of 1 × 10^6^ cells/mL.

### 2.8. Isolation and Culture of Mesenteric Lymph Nodes (MLNs) Cells

MLNs were removed and transferred to ice cold sterile Hank's balanced salt solution. The nodes were disrupted and passed through a nylon mesh (70 *μ*m pore size). Cells were then incubated in RPMI 1640 with 10% FCS and 100 IU/mL penicillin/streptomycin at a concentration of 1 × 10^6^ cells/mL for 48 h in the presence of anti-CD3 and anti-CD28 antibodies (eBioscience, San Diego, CA). Cytokine production in culture supernatants was determined by enzyme-linked immunosorbent assay (ELISA).

### 2.9. ELISA

Frozen colonic samples were homogenized mechanically in lysis buffer. Homogenized tissue samples were centrifuged at 18,300 ×g at 4°C for 30 min. Homogenized tissue or cell culture supernatants were collected, and cytokine levels of TNF-*α*, IL-1*β*, IL-12, IFN-*γ*, IL-4, IL-13, IL-5, IL-6, IL-17, and IL-10 in the supernatant were determined using ELISA kits, according to the manufacturer's instructions (R&D Systems, Minneapolis, MN).

### 2.10. Real-Time Polymerase Chain Reaction (PCR)

Total RNA was extracted using TRIzol reagent (Invitrogen). 1 *μ*g total RNA was then reverse transcribed into first-strand cDNAs and was synthesized with M-MLV reverse transcriptase. Primer sequences are listed as follows: IFN-*γ* forward 5′-AGGATGCATTCATGAGCATCGCC-3′ and reverse 5′-TCAGCACCGACTCCTTTTCCGCT-3′; IL-12 forward 5′-AGTGTAACCAGAAAGGTGCGTTC-3′ and reverse 5′-CCTGCAGGGTACACATGTCCATT-3′; IL-4 forward 5′-CGGCAACAAGGAACACCACGGA-3′ and reverse 5′-AGCGTGGACTCATTCACGGTGC-3′; IL-13 Forward 5′-CTGCAGTCCTGGCTCTCGC-3′ and reverse 5′-CTTTTCCGCTATGGCCACTG-3′; IL-5 forward 5′-ACGATGAGGCTTCCTGTTCC-3′ and reverse 5′-TTCCATTGCCCACTCTGTAC-3′; IL-6 forward 5′-GTGCAATGGCAATTCTGATTGTA-3′ and reverse 5′-CTAGGGTTTCAGTATTGCTCTGA-3′; IL-17 forward 5′-AGCTCCAGAAGGCCCTCAGACTA-3′ and reverse 5′-CAGGACCAGGATCTCTTGCTGGA-3′; IL-10 forward 5′-CCAAGCCTTGTCAGAAATGATCA-3′ and reverse 5′-CTCATTCATGGCCTTGTAGACAC-3′; T-bet forward 5′-AACCAGTATCCTGTTCCCAGC-3′ and reverse 5′-TGTCGCCACTGGAAGGATAG-3′; GATA-3 forward 5′-CAGTCCGCATCTCTTCAC-3′ and reverse 5′-TAGTGCCCAGTACCATCTC-3′; ROR*γ*t forward 5′-AGTAGGCCACATTACACTGCT-3′ and reverse 5′-GACCCACACCTCACAAATTGA-3′; Foxp3 forward 5′-GTACAGCCGGACACACTGC-3′ and reverse 5′-GCTGACTTCCAAGTCTCGTGT-3′. Mean relative gene expression was calculated using the 2^−ΔΔCt^ formula, as described previously [18].

### 2.11. Western Blotting

Proteins were extracted in complete radioimmunoprecipitation lysis buffer (RIPA) supplemented with protease inhibitor cocktails (Roche Diagnostics). Protein concentrations of the samples were determined using a bicinchoninic acid assay kit (Thermo Scientific, Bremen, Germany), and the samples were then boiled for 10 minutes. Equal amounts of protein (50 *μ*g) were separated by SDS-PAGE and electrophoretically transferred onto polyvinylidene fluoride (PVDF) membranes (Millipore Corp., Bedford, MA). After blocking by incubation in 5% nonfat dry milk for 1 h at room temperature, the membranes were incubated overnight with primary antibodies recognizing *β*-actin, T-bet, GATA-3, ROR-*γ*t, and Foxp3 (Santa Cruz, CA, USA) at 4°C with gentle shaking. After washing three times with TBST, the blots were incubated with horseradish peroxidase- (HRP-) conjugated secondary antibodies (Cell Signaling Technology) for 1 h. The blots were washed three times, visualized using the enhanced chemiluminescence (ECL) detection system (Amersham Biosciences, Buckinghamshire, UK), and quantified with the Quantity One System (Bio-Rad).

### 2.12. Flow Cytometry and Intracellular Staining

All antibodies used for cell labeling were purchased from eBioscience (San Diego, CA, USA). For measurements of intracellular cytokines, cells were stimulated with PMA (1 *μ*g/mL) and ionomycin (50 *μ*g/mL) in the presence of monensin (0.1 mg/mL) at 37°C and 5% CO_2_ for 5 h. Cells were then washed in PBS and surface-labeled with fluorescein isothiocyanate- (FITC-) conjugated anti-CD4. After fixation and permeabilization, the cells were stained with phycoerythrin-cyanin 7- (PE-Cy7-) conjugated anti-IL-17, PerCP-Cy5-conjugated anti-IFN-*γ*, and allophycocyanin- (APC-) conjugated anti-IL-4. For analysis of Treg cells, they were aliquoted into tubes without PMA and ionomycin stimulation, and surface staining was performed with FITC conjugated anti-CD4 and PE conjugated anti-CD25 antibodies. Then cells were fixed and permeabilized with Fix/Perm solution, and intracellular staining was performed with APC-conjugated anti-Foxp3. The stained cells were analyzed using a FACS Canto cytometer (BD Bioscience), and the data were analyzed with FlowJo (TreeStar).

### 2.13. Statistics

Results are presented as the mean ± SD. Differences between two groups were examined using unpaired Student's *t*-tests. For analyzing multiple groups, a one-way ANOVA was used. The clinical activity score of colitis as well as macroscopic and histological scores was statistically analyzed using the Kruskal-Wallis nonparametric test, followed by the Mann-Whitney *U*-test, to compare the results of the different groups. *P* values < 0.05 were considered significant.

## 3. Results

### 3.1. HQT Ameliorates TNBS-Induced Colitis in a Dose-Dependent Manner

**Figure 1:**
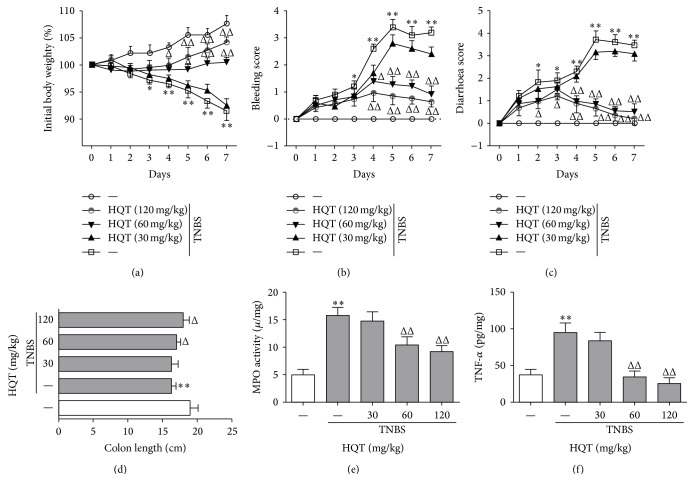
HQT ameliorates TNBS-induced colitis in a dose-dependent manner. Various doses of Huangqin-Tang decoction (HQT) (30–120 mg/kg) were administered following the 2,4,6-trinitrobenzenesulfonic acid (TNBS) enema and on the next 2 days. (a) Body weight changes (percentage of original body weight), (b) bleeding score, and (c) diarrhoea score were scored daily. (d) Rats were sacrificed on day 7 to measure colon length. (e) Myeloperoxidase (MPO) activity was assessed in colon homogenates as described in Section 2. (f) The production of tumor necrosis factor-*α* (TNF-*α*) in the colon was determined by enzyme-linked immunosorbent assay (ELISA). Results represent the mean ± SD from eight mice per group. ^*^ *P* < 0.05, ^**^ *P* < 0.001 versus the control group. ^Δ^ *P* < 0.05, ^ΔΔ^ *P* < 0.001 versus TNBS-treated rats.

Intrarectal administration of TNBS has long been used as an alternative model for the induction of acute colitis [19]. To assess whether HQT exerts a protective role during colitis, HQT was first evaluated in a dose-response study of TNBS-induced colitis at 30 mg/kg, 60 mg/kg, and 120 mg/kg concentrations. Rats were treated with HQT for 7 consecutive days starting on day 2 after induction of TNBS colitis. As expected, rats given TNBS developed severe colitis, characterized by a profound and sustained weight loss, bleeding, and diarrhoea. Here, HQT treatment rapidly reversed the lost body weight and decreased the extent of the bleeding and diarrhoea scores in a dose-dependent manner, with both parameters reaching significance in the 60 and 120 mg/kg treatment groups (Figures 1(a)–1(c)). Consistently, this treatment also significantly prevented colon shortening and decreased MPO activity (Figures 1(d) and 1(e)). Furthermore, TNF-*α* in colon culture supernatants was significantly lower in HQT-treated rats compared to rats treated with TNBS alone (Figure 1(f)). Since treatment with 120 mg/kg HQT proved to be most effective in the amelioration of colitis, this concentration was used in further experiments. Collectively, these results demonstrate that HQT is effective in protecting against acute TNBS-induced colitis in a dose-dependent manner.

### 3.2. The Anti-Inflammatory Potency of HQT is Superior to Mesalazine in the TNBS-Induced Colitis Model

**Figure 2:**
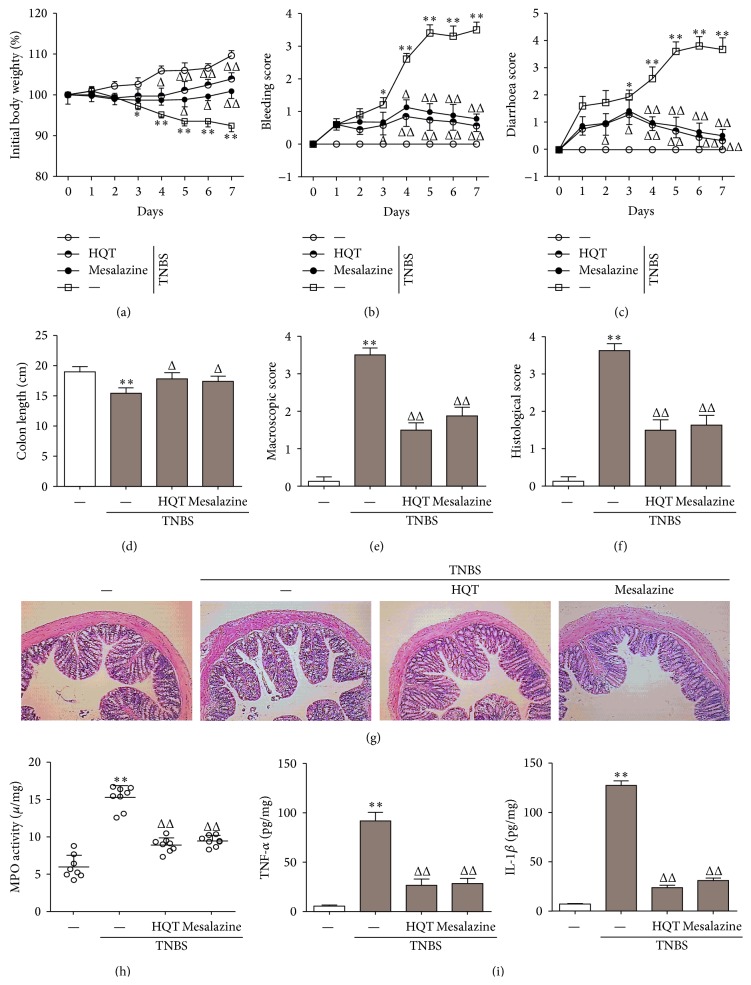
HQT protects against TNBS-induced colitis in a manner equal to mesalazine. Rats with TNBS-induced colitis were treated with HQT (120 mg/kg) or mesalazine (100 mg/kg). (a) Body weight changes (percentage of original body weight), (b) bleeding score, and (c) diarrhoea score were scored daily. (d) Rats were sacrificed on day 7 to measure colon length. (e) Macroscopic score was evaluated on day 7. (f) Histological score in colons. (g) Colon sections from each group rat were stained with H&E (original magnification, 100x). (h) MPO activity was assessed in colon homogenates, as described in Section 2. (i) The production of TNF-*α* and interleukin- (IL-) 1*β* in the colon was determined by ELISA. Results represent the mean ± SD from eight mice per group. ^*^ *P* < 0.05, ^**^ *P* < 0.001 versus the control group. ^Δ^ *P* < 0.05, ^ΔΔ^ *P* < 0.001 versus TNBS-treated rats.

Oral administration of mesalazine is the first-line approach to induce and maintain clinical remission in patients with mild-to-moderate UC or CD [20]. To assess the anti-inflammatory potency of HQT in TNBS-induced colitis, the effect of HQT (120 mg/kg) and mesalazine (100 mg/kg) was directly compared. When studying the clinical course of the disease, TNBS-treated rats suffered the most body weight loss from day 3 onward (Figure 2(a)). Starting at day 4, treatment with HQT or mesalazine resulted in a higher weight gain compared to animals treated with TNBS alone (Figure 2(a)). Simultaneously, bleeding and diarrhoea scores of TNBS-treated rats became significantly worse compared to those of controls. In contrast, such changes were markedly improved by HQT or mesalazine treatment (Figures 2(b) and 2(c)).

To further assess the severity of colitis, colon length was measured in each group of rats. Colons of rats treated with TNBS alone were on average 10% shorter than those of rats subjected to additional treatment with HQT or mesalazine (Figure 2(d)). This inflammatory phenotype was further evidenced by the gross and microscopic appearances of the colon. Consistent with the clinical parameters discussed above, treatment with HQT or mesalazine significantly ameliorated the macroscopic scores compared to rats treated with TNBS alone (Figure 2(e)). Histological sections revealed no substantial disease in activity in control rats, whereas in TNBS-treated rats, severe inflammation could be detected, including more infiltrating inflammatory cells and significantly more ulceration. However, colon section from HQT or mesalazine groups showed a marked reduction in the tissue disruption, mucosal ulcerations, and mononuclear cell infiltration (Figure 2(g)). Furthermore, histological scoring revealed that HQT or mesalazine treatment reduced the severity of TNBS-induced colitis (Figure 2(f)). Consistent with these histological changes, TNBS significantly increased colonic MPO activity. In contrast, all HQT-treated rats as well as mesalazine-treated rats presented decreased colonic MPO activity compared to rats treated with TNBS alone (Figure 2(h)).

Furthermore, inflammatory cytokine expression levels, including those of TNF-*α* and IL-1*β*, were also clearly induced in TNBS-treated rats compared to control rats. Administration of HQT or mesalazine prevented the induction of these inflammatory cytokines (Figure 2(i)), suggesting that HQT treatment might have broad anti-inflammatory activity. Together, these results clearly indicate that HQT plays a therapeutic role and is superior to mesalazine in resolving the inflammatory response following TNBS-induced injury of the colon.

### 3.3. Distinct Effects of HQT on the Frequencies of Th1, Th2, Th17, and Treg Cells in the TNBS-Induced Colitis Model

**Figure 3:**
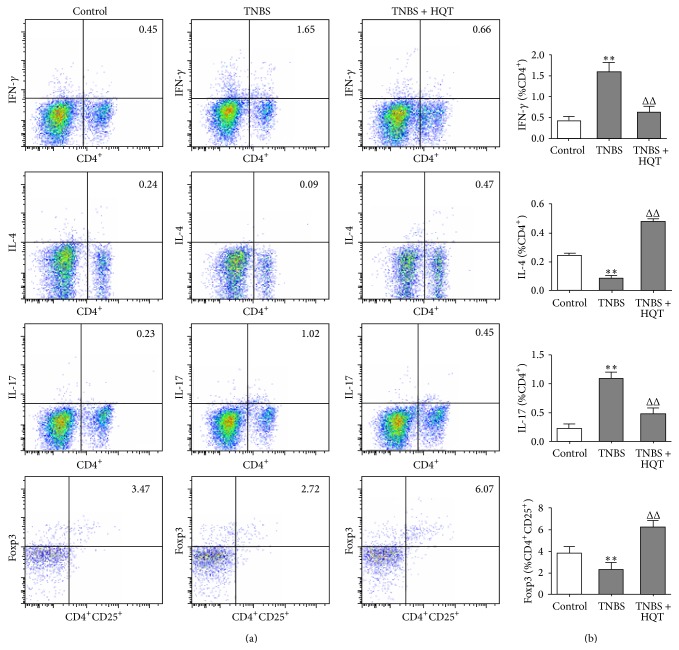
HQT regulates frequencies of Th1, Th2, Th17, and Treg in the TNBS-induced colitis model. Rats with TNBS-induced colitis were treated with or without HQT (120 mg/kg) and analyzed 7 days after treatment. Lamina propria mononuclear cells (LPMCs) were isolated from each group and subjected to intracellular IFN-*γ*, IL-4, IL-17, and Foxp3 staining. (a) The frequency of T helper 1 (Th1) (CD4^+^IFN-*γ* ^+^), Th2 (CD4^+^IL-4^+^), Th17 (CD4^+^IL-17^+^), and regulatory T (Treg) (CD4^+^CD25^+^Foxp3^+^) was determined by flow cytometry. Numbers represent the percentages of IFN-*γ*, IL-4, IL-17A-expressing CD4^+^ T cells, and Foxp3-expressing CD4^+^CD25^+^ T cells in each quadrant. (b) Quantitative analysis of the frequency and total number of Th1, Th2, Th17, and Treg in LPMCs. Results represent the mean ± SD from eight mice per group. ^*^ *P* < 0.05, ^*^ *P* < 0.001 versus the control group. ^Δ^ *P* < 0.05, ^ΔΔ^ *P* < 0.001 versus TNBS-treated rats.

As studies have demonstrated that the Th1, Th2, Th17, and Treg CD4^+^ T cells subsets play distinct roles in the control and development of IBD [2], we hypothesized that HQT may differentially contribute to the development of these CD4^+^ T cells subsets. Using flow cytometry, we determined the proportions of Th1, Th2, Th17, and Treg cells among LPMCs in the TNBS-induced colitis model. LPMCs cells were treated with phorbol myristate acetate-ionomycin, stained for cell surface CD4, and intracellularly stained for interferon- (IFN-) *γ*, IL-4, and IL-17 to detect Th1, Th2, and Th17 cells, respectively. As shown in Figures 3(a) and 3(b), we observed that there were marked increases in the numbers of IFN^+^ and IL-17^+^ CD4^+^ T cells after TNBS challenge, while the IL-4^+^ CD4^+^ T cell decreased. The proportions of Th1 and Th17 cells in the HQT-treated TNBS group were significantly lower than those in the TNBS group. In contrast to the Th1 and Th17 cells, we observed a significantly higher frequency of IL-4-producing Th2 cells in LPMCs of rats that were treated with HQT, compared with rats treated with TNBS alone.

For analysis of Treg cells, LPMCs were surface-labeled with CD4 and CD25 antibodies, followed by intracellular staining with Foxp3. Results show that HQT treatment increased the CD4^+^ CD25^+^ Foxp3^+^ Treg levels amongst LPMCs. Thus, our results clearly indicate that the ability of HQT to ameliorate colitis was associated with an expansion of Th2 and Treg cells and a reduction of Th1 and Th17 cells among LPMCs.

### 3.4. HQT Regulates Th1-, Th2-, Th17-, and Treg-Related Cytokine Production in the TNBS-Induced Colitis Model

**Figure 4:**
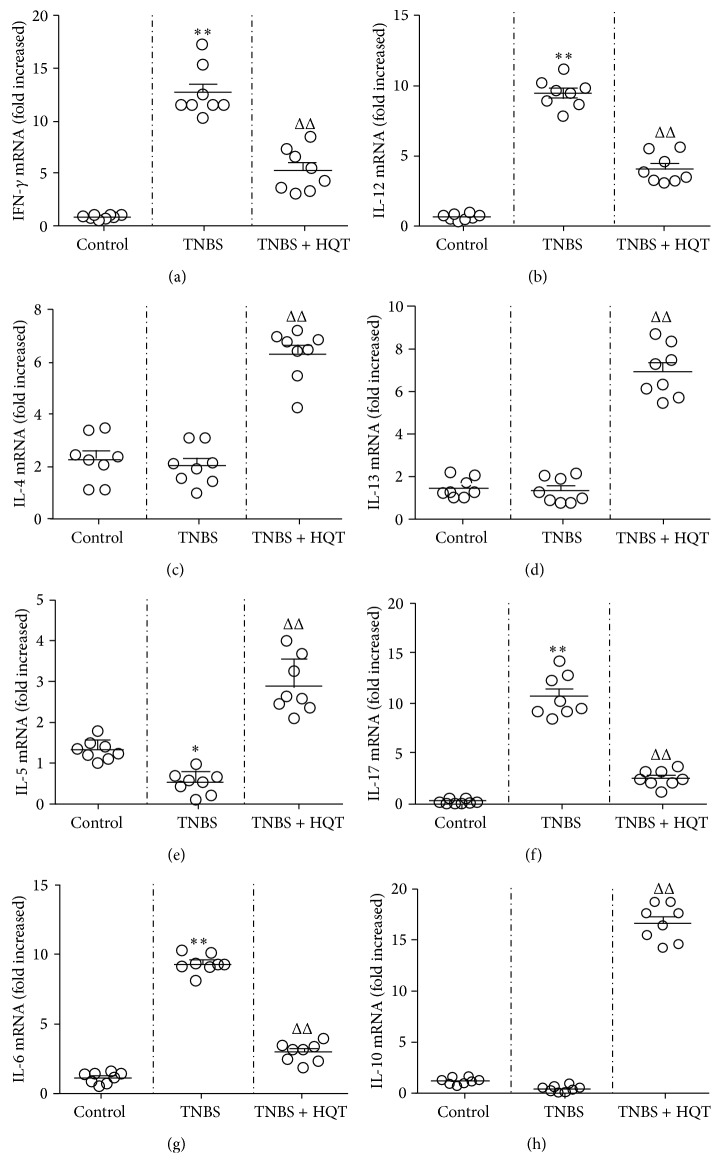
HQT regulates mRNA expression of Th1-, Th2-, Th17-, and Treg-related cytokines in the TNBS-induced colitis model. Rats with TNBS-induced colitis were treated with or without HQT (120 mg/kg). Total mRNA was extracted from colonic tissue to analyze the expression of Th1-related cytokines (a) IFN-*γ* and (b) IL-12; Th2-related cytokines (c) IL-4, (d) IL-13, and (e) IL-5; Th17-related cytokines (f) IL-17A and (g) IL-6; Treg-related cytokines (h) IL-10 by real-time polymerase chain reaction (PCR). Results represent the mean ± SD from eight mice per group. ^*^ *P* < 0.05, ^**^ *P* < 0.001 versus the control group. ^Δ^ *P* < 0.05, ^ΔΔ^ *P* < 0.001 versus TNBS-treated rats.

**Figure 5:**
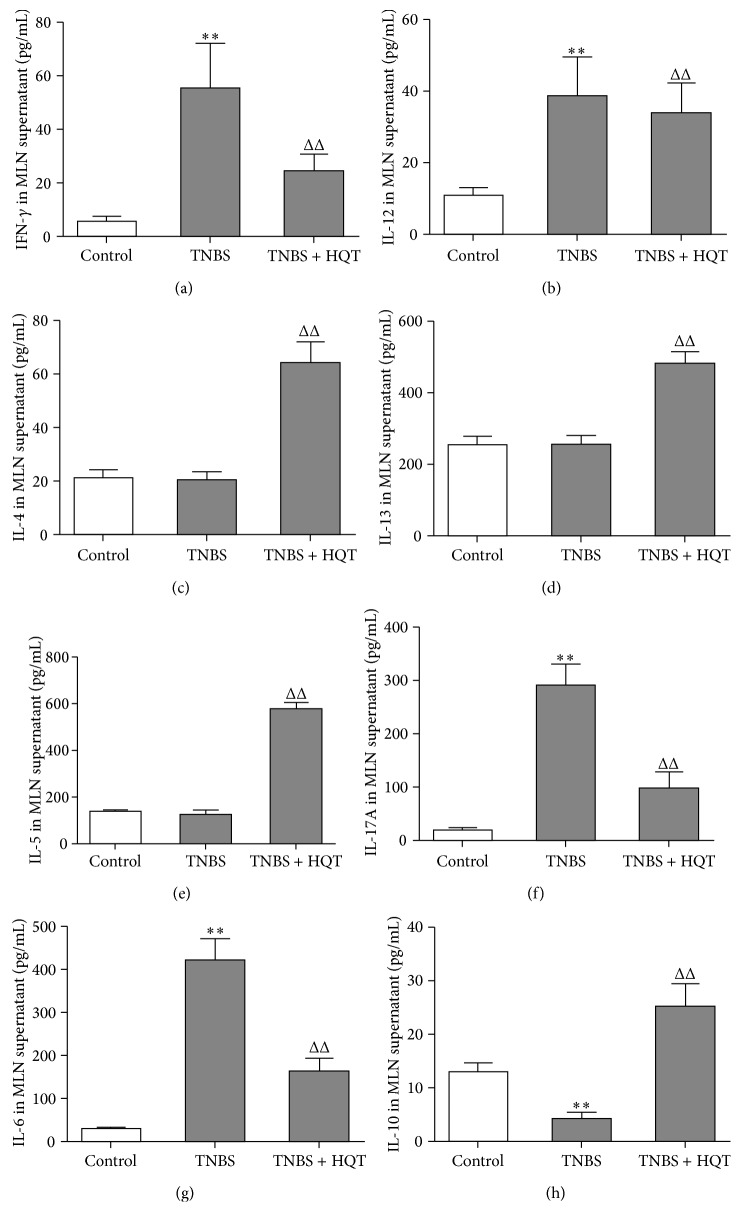
HQT regulates protein levels of Th1-, Th2-, Th17-, and Treg-related cytokines in the TNBS-induced colitis model. Rats with TNBS-induced colitis were treated with or without HQT (120 mg/kg). Mesenteric lymph nodes (MLNs) cells from each group were stimulated with anti-CD3/CD28 antibodies and the cultural supernatants were harvested, followed by ELISA analysis of cytokines indicated above (pg/mL). Th1-related cytokines (a) IFN-*γ* and (b) IL-12; Th2-related cytokines (c) IL-4, (d) IL-13, and (e) IL-5; Th17-related cytokines (f) IL-17A and (g) IL-6; Treg-related cytokines (h) IL-10 were measured. Results represent the mean ± SD from eight mice per group. ^*^ *P* < 0.05, ^**^ *P* < 0.001 versus the control group. ^Δ^ *P* < 0.05, ^ΔΔ^ *P* < 0.001 versus TNBS-treated rats.

To determine the effect of HQT on driving Th cell responses in rats with TNBS-induced colitis, we further measured the production of signature cytokines that are critical for the differentiation of Th subsets in MLNs and colonic tissue. Our results revealed that TNBS-treated rats exhibited an aberrant cytokine pattern, characterized by mRNA overexpression of Th1 and Th17 signature cytokines, including IFN-*γ*, IL-12, IL-17, and IL-6, and this increase was significantly decreased by administration of HQT (Figure 4). Moreover, total protein extracted from MLNs was analyzed by ELISA. Similarly, HQT significantly downregulated cytokine levels of IFN-*γ*, IL-12, IL-17, and IL-6 in TNBS-treated rats (Figure 5). Contrary to the decreased Th1- and Th17-associated cytokines, the protein and mRNA expression in MLNs and colonic tissue showed increased production of Th2- and Treg-associated cytokines IL-4, IL-5, IL-13, and IL-10 in HQT-treated rats (Figures 4 and 5). Taken together, these results indicate that HQT administration inhibits Th1 and Th17 responses but promotes Th2 and Treg responses in TNBS-induced colitis.

### 3.5. Effect of HQT on Th1, Th2, Th17, and Treg Transcription Factors in TNBS-Induced Colitis

**Figure 6:**
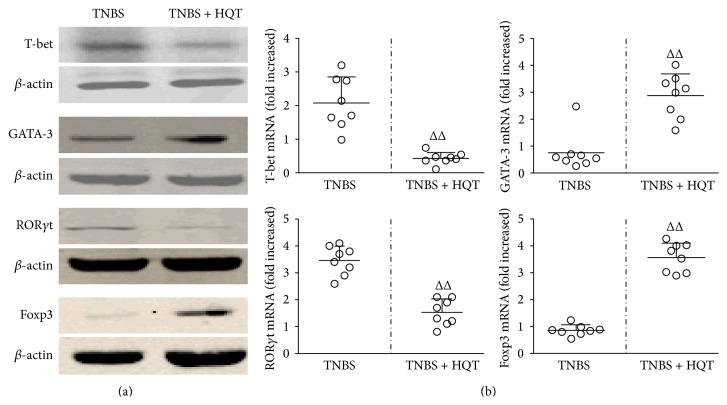
HQT regulates protein and mRNA levels of T-bet, GATA-3, ROR-*γ*t, and Foxp3 in the TNBS-induced colitis model. Rats with TNBS-induced colitis were treated with or without HQT (120 mg/kg). (a) Whole colon tissue homogenates collected at 7 days after HQT treatment were examined for T-bet, GATA-3, ROR*γ*t, and Foxp3 by western blot analysis. Each lane corresponds to an individual mouse. (b) Distal colons collected at day 7 after HQT treatment were used to isolate RNA for expression analysis of T-bet, GATA-3, ROR-*γ*t, and Foxp3 by real-time PCR. Results represent the mean ± SD from eight mice per group. ^Δ^ *P* < 0.05, ^ΔΔ^ *P* < 0.001 versus TNBS-treated rats.

To understand the molecular mechanism by which HQT affects CD4^+^ T cell subsets, we determined the expression levels of the nuclear transcription factors of these subsets using western blot and real-time PCR. We found that HQT treatment enhanced the expression of Foxp3 and GATA-3, but it reduced the expression of T-bet and ROR-*γ*t, both at the protein and gene expression levels (Figures 6(a) and 6(b)). Taken together, we demonstrate a crucial role for HQT that prevent the development of intestinal inflammation and maintain intestinal immune homeostasis.

## 4. Discussion

In the present study, we illustrated an important role for HQT in inhibiting TNBS-mediated intestinal inflammation. We first demonstrated that administration of HQT at doses of 30–120 mg/kg significantly attenuated colitis in a dose-dependent manner. In addition, administration of 120 mg/kg HQT was significantly more potent than mesalazine (100 mg/kg) in ameliorating TNBS-induced colitis. Moreover, mechanistic studies indicate that the anti-inflammatory effects of HQT are involved in restraining Th1 and Th17 responses, while enhancing Th2 and Treg responses, to TNBS challenge in this murine colitis model. Therefore, our report unveiled for the first time, to our knowledge, an important role for HQT in anti-inflammatory and immunomodulatory effect in IBD.

To evaluate the effect of HQT, we used the well-established model of TNBS-induced colitis in rats which has resemblance to CD [21]. In the present study, HQT efficiently and dose-dependently improved TNBS-induced colitis. It caused attenuation of weight loss, diarrhoea, and bleeding scores while preserving colonic length and reducing MPO activity, a marker of tissue neutrophil activation [22]. As expected, TNBS treatment markedly increased TNF-*α* protein expression in the colon, and that increase was reduced significantly and dose-dependently by HQT treatment. Consequently, treatment with 120 mg/kg HQT was the most effective with respect to the amelioration of colitis and was used in our experiments.

Mesalazine is one of the most commonly prescribed anti-inflammatory drugs that is used to treat IBD [23]. Studies have demonstrated the significant and comparable protection of mesalazine on experimental colitis induced by TNBS [24]. Here, the effects of HQT (120 mg/kg) and mesalazine (100 mg/kg) were directly compared in experimental colitis. We showed that HQT as well as mesalazine dramatically inhibited weight loss, bleeding, and diarrhoea score while preserving colonic length. In addition to exerting such beneficial clinical effects, treatment with HQT and mesalazine also resulted in macroscopic and microscopic amelioration of intestinal inflammation, consistent with reduced MPO activity. Elevated levels of proinflammatory cytokines, such as TNF-*α* and IL-1*β*, were demonstrated during the development of IBD and experimental colitis. TNF-*α* monoclonal antibodies have been shown to dramatically decrease signs and symptoms of IBD and subsequently are key potential therapeutic agents [25, 26]. In the present study, we further demonstrate that local TNF-*α* and IL-1*β* expressions are decreased after HQT or mesalazine treatment in rats with TNBS-induced colitis. Our results suggest that daily HQT administration significantly inhibited the progression of colitis, yielding a protective effect equal to or even greater than that of mesalazine.

Furthermore, our work highlights the fact that HQT uniquely interacts with the host immune system to exact its immunoregulatory potency. More recently, studies have highlighted the roles of T cell subsets in IBD [27]. Classical Th1/Th2 pathways are thought to play a critical role in IBD pathogenesis. It is widely accepted that TNBS-induced colitis is mediated by a dominant Th1 immune response and a deficiency of Th2 responses [28, 29]. Moreover, recent studies have highlighted a key pathogenic role of Th17 cells, and increased numbers of Th17 cells have been found in IBD patients and animal models [30–32]. On the contrary, Treg cells are key players in maintaining immune homeostasis, and they regulate immune responses to allergens by preventing excessive inflammatory responses [33]. Recent studies demonstrated a decrease in Treg cells number in IBD patients and animal models [34–36]. Here, we found that, in the progression of TNBS-induced colitis, treatment with HQT significantly decreased the percentage of Th1 and Th17 cells among LPMCs. Simultaneously, the numbers of Th2 and Treg cells markedly increased when compared with the TNBS-induced colitis group. This implies that the rehabilitating effect of HQT in IBD works by restoring the balance between CD4^+^ T cells subsets.

Homeostasis of distinct Th cell subset-derived cytokines plays a crucial role in mediating intestinal inflammation in IBD. Studies have shown that Th1-related cytokines (IFN-*γ* and IL-12) and Th17-associated cytokines (IL-17A, IL-21, and IL-23) are markedly increased in CD, while in UC there is increased production of the Th2 cytokines (IL-5, IL-13, and IL-4) [37]. IL-10 is an important anti-inflammatory cytokine that can be secreted by Treg cells, and IL-10 defects cause spontaneous colitis in mice [38]. In addition, numerous studies have shown that a change in the cytokine profile from Th1 and Th17 to Th2 and Treg could ameliorate Th1/Th17-mediated diseases, such as CD and TNBS-induced colitis [29, 39, 40]. ELISA and real-time PCR methods were used in this study to detect the expression of cytokines related to the different CD4^+^ T cells subsets. In agreement with a suppression of Th1 and Th17 numbers amongst LPMCs, HQT-treated rats exhibited defective production of Th1- and Th17-associated cytokines. Nevertheless, increased production of Th2- and Treg-associated cytokines were observed in TNBS-treated rats, suggesting that HQT significantly improved inflammation and ameliorated disease in TNBS-treated rats, associated with a shift from a Th1 and Th17 profile to a Th2 and Treg immunological profile.

Because transcription factors are crucial for T-cell differentiation, we also examined lineage-specific transcription factors. The Th1 transcription factor T-bet plays a critical role in the development of Th1-driven colitis due to the high expression levels of IFN-*γ* [41], while GATA3 is an essential master regulator of Th2 cells for the induction of IL-4, IL-5, and IL-13 [5]. Although Th17 and Treg cells share a common requirement for TGF-*β* in their differentiation, their distinct transcriptional regulators ROR-*γ*t and Foxp3 are necessary, respectively [31]. ROR-*γ*t directs Th17 differentiation and induces the production of IL-17 [42], and Foxp3 dominates Treg formation and production of regulatory cytokines, such as TGF-*β* and IL-10 [43]. Our results showed that the colonic protein and mRNA expression levels of T-bet and ROR-*γ*t significantly decreased but GATA-3 and Foxp3 expressions were enhanced in colon after HQT treatment in colitis rats. These data indicate that HQT plays a significant role during IBD development in establishing the homeostasis of distinct Th cell subsets in response to TNBS challenge.

In this study, we presented evidence that HQT-treatment elicits a strong Th2 and Treg response in TNBS-induced colitis. It has been reported that Th1 and Th2 cells and the cytokines they release are often mutually antagonistic, and a change in the cytokine profile from Th1 to Th2 could ameliorate Th1-mediated disease [29, 44]. Consistent with these findings, a strong Th2 response successfully counteracts Th1/Th17-mediated colitis [45], suggesting that the role of Th2 in intestinal inflammation may be protective in TNBS-induced colitis. Additionally, Treg cells have been reported to repress the activity of other T cell subsets to induce an anti-inflammatory response [46]. It is therefore conceivable that the protective effect of HQT on TNBS-induced colitis might be explained by its capability to induce Treg cells and rebalance CD4^+^ T cell subsets. Although there were no significant side effects associated with HQT treatment in our study, more detailed studies are necessary to prove its immunomodulatory effect in different models of colitis.

In conclusion, our results indicate that HQT plays an important role in the regulation of intestinal immune responses in TNBS-induced colitis by downregulating effector phenotype of Th1 and Th17 cells, while promoting Th2 and Treg responses. Thus, using HQT, a Chinese medicinal formulation, to regulate immune homeostasis may offer a promising alternative to our current therapeutic strategy for IBD.

## Abbreviations

HQT: Huangqin-Tang decoction
IBD: Inflammatory bowel disease
TNBS: 2,4,6-Trinitrobenzenesulfonic acid
LPMCs: Lamina propria mononuclear cells
MLNs: Mesenteric lymph nodes
MPO: Myeloperoxidase
Tregs: Regulatory CD4^+^ T cells.
